# Relative Validity and Reproducibility of a Semi-quantitative Food Frequency Questionnaire to Assess Nutrient Intakes of PERSIAN Cohort Participants: Comparisons to 24-Hour Dietary Recalls and Selected Biomarkers

**DOI:** 10.34172/aim.34573

**Published:** 2025-09-01

**Authors:** Sareh Eghtesad, Maryam Sharafkhah, Azita Hekmatdoost, Alireza Ostadrahimi, Mojtaba Farjam, Alireza Vakilian, Yahya Pasdar, Motahareh Kheradmand, Fariba Shahraki-Sanavi, Amir Houshang Mehrparvar, Amaneh Shayanrad, Zahra Mohammadi, Monireh Sadat Seyyedsalehi, Walter C. Willett, Reza Malekzadeh, Hossein Poustchi

**Affiliations:** ^1^Liver and Pancreatobiliary Diseases Research Center, Digestive Diseases Research Institute, Tehran University of Medical Sciences, Tehran, Iran; ^2^Department of Clinical Nutrition & Dietetics, National Nutrition & Food Technology Research Institute, Shahid Beheshti University of Medical Sciences, Tehran, Iran; ^3^Nutrition Research Center, Tabriz University of Medical Sciences, Tabriz, Iran; ^4^Noncommunicable Diseases Research Center, Fasa University of Medical Sciences, Fasa, Iran; ^5^Neurology Department, School of Medicine, Non-Communicable Diseases Research Center, Rafsanjan University of Medical Sciences, Rafsanjan, Iran; ^6^Research Center for Environmental Determinants of Health (RCEDH), Nutritional Sciences Department, Kermanshah University of Medical Sciences, Kermanshah, Iran; ^7^Health Sciences Research Center, Mazandaran University of Medical Sciences, Sari, Iran; ^8^Health Promotion Research Center, Zahedan University of Medical Sciences, Zahedan, Iran; ^9^Industrial Diseases Research Center, Shahid Sadoughi University of Medical Sciences, Yazd, Iran; ^10^Cancer Research Center, Cancer Institute, Tehran University of Medical Sciences, Tehran, Iran; ^11^Department of Medical and Surgical Sciences, University of Bologna, Bologna, Italy; ^12^Department of Nutrition, Harvard T.H. School of Public Health, Boston, MA, USA; ^13^Department of Epidemiology, Harvard T.H. School of Public Health, Boston, MA, USA; ^14^Digestive Diseases Research Center, Digestive Diseases Research Institute, Tehran University of Medical Sciences, Tehran, Iran

**Keywords:** FFQ, Food frequency questionnaire, Nutrients, PERSIAN cohort, Reproducibility, Validity

## Abstract

**Background:**

Food frequency questionnaires (FFQs) are widely used in epidemiologic studies to assess the dietary intake of individuals. In this study, we evaluated the validity and reproducibility of the FFQ used in the Prospective Epidemiological Research Studies in IrAN (PERSIAN) for assessing nutrient intakes.

**Methods:**

Individuals (n=978) from seven PERSIAN cohort centers participated in this study; an initial FFQ was completed for each person upon enrollment (FFQ1), followed by two 24-hour dietary recalls (24HR) each month, for twelve months, and finally, another FFQ at the end of the study (FFQ2). Serum and 24-hour urine (24H-U) samples were also collected each season, and selected biomarkers were measured. To assess validity, correlation coefficients between the 24HRs and the FFQs were obtained. The triad method was used to compare the biomarkers to the FFQs. Correlations between FFQ1 and FFQ2 were evaluated to assess the reproducibility of the questionnaire.

**Results:**

Correlations obtained for energy and macronutrients, in comparing FFQ1 and FFQ2 to the 24HRs were 0.57,0.63 (energy), 0.56,0.62 (protein), 0.51,0.55 (lipids) and 0.42,0.51 (carbohydrates), respectively. Moderate (0.4‒0.6) and high (>0.6) correlations were seen for micronutrients, with only vitamins B6 and B12 being poorly correlated (<0.4). Validity coefficients obtained for urinary protein and sodium, as well as serum folate and selected fatty acids were acceptably above 0.4. Reproducibility correlations ranged from 0.18 (alpha-linoleic acid) to 0.78 (selenium), with 19 of the 30 evaluated nutrients showing high and 2 showing poor correlations.

**Conclusion:**

Overall, the PERSIAN Cohort FFQ is acceptable to rank individuals based on their nutrient intakes.

## Introduction

 Dietary intake is an important modifiable risk factor of non-communicable diseases (NCDs) such as cardio-cerebrovascular diseases, type II diabetes and cancers, among others. NCDs account for over 80% of premature mortality in Iran,^1,2^ with 16.5% of total deaths being attributed to dietary risk factors, independent of age and gender.^3,4^ Therefore, a reliable means of collecting dietary information has a significant impact on identifying accurate diet-NCD associations, which can in turn, help the development of food related policies to prevent and control NCDs.

 The food frequency questionnaire (FFQ) is widely used in epidemiologic studies to assess dietary intake by ranking individuals based on their food and nutrient intake or by assessing overall dietary patterns in a population.^5^ FFQs evaluate long-term dietary intake as opposed to other dietary methods such as diet records or 24-hour dietary recalls (24HR), which makes them suitable for assessing diet-NCD associations, as NCDs also take long to develop and are affected by long-term habits captured by the FFQ.

 The FFQ used in the PERSIAN (Prospective Epidemiological Research Studies in IrAN)Cohort Study (the largest prospective epidemiological cohort in Iran aimed at identifying the burden of NCDs and their risk factors) has been previously validated at the food group^6^ and dietary patterns levels (being published). While it is generally accepted that the overall dietary pattern, and not necessarily single nutrients, affect NCD development, it is still important to study nutrient intake, as well, especially since the introduction of Nutrigenomics, and new insights on how nutrients can play important roles in DNA stability and gene expression, affecting the prevention or development of NCDs.^7^ Previous studies have shown vitamins A and D, as well as fatty acids, to have direct actions in gene transcription, and other compounds such as resveratrol, polyphenols, tocotrienols and phytochemicals to play key roles associated with inflammation, cardiovascular diseases and cancers.^8-12^ We therefore decided to assess the validity of the PERSIAN Cohort FFQ for energy and nutrient intake, as well, in order to verify whether the data it provides can be used to assess the role nutrients may have in NCD development and in future genetic studies.

 This manuscript aims to present the validity and reproducibility of the PERSIAN Cohort FFQ for nutrient intake, in comparison to 24HRs, as well as selected serum and urine biomarkers.

## Materials and Methods

 This validation study was conducted as part of the pilot phase of the PERSIAN Cohort Study. The rationale, design and objectives of the PERSIAN Cohort have been previously explained,^1,13^ but briefly, this cohort includes 163,000 men and women 35‒70 years of age, from 18 geographically and ethnically distinct areas of Iran, aiming to determine the burden of common NCDs as well as the risk factors associated with them. Many questionnaires evaluating various environmental, social and other exposures were completed for participants at baseline data collection from 2014‒2019, including an FFQ to assess dietary intake. Individuals are then followed each year and occurrence of the study’s outcomes, such as NCD incidence or death, are recorded.

###  Study Locations and Participants

 The FFQ validation study was conducted from 2015‒2017 in seven of the PERSIAN Cohort centers: Fasa, Azar, Tabari, Yazd, Zahedan, Rafsanjan and Kermanshah. While the duration of data collection for each participant entering the validation study was one year, the study stretched over approximately three years because we attempted to include individuals from various ethnic populations with different cultural and dietary habits in the validation study to ensure that the FFQ is able to capture the dietary habits of the different participants included in the PERSIAN Cohort, and the pilot phase at the various cohort centers, and hence the validation study at that center, started at different times.

 We invited 1,260 individuals (180 from each of the seven cohort centers) who enrolled in the PERSIAN Cohort to also participate in the validation study. Our sample collection relied on invitations in the main cohort and when the desired sample size was reached at each center, enrollment for the validation study ceased. Of those invited, 1,097 individuals agreed to participate in the study. Our overall sample size exceeds typical recommendations for a validation study (100-200 individuals),^5^ but our goal in having this large sample size was to include an adequate number of individuals from each location in order to have adequate representation of their dietary habits.

###  Data collection: Dietary Assessment and Biological Sampling

**Figure 1:**
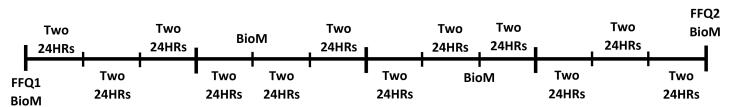
Timeline of Data and Biological Sample Collection for Individuals Over the Study’s 12-Month Duration. Each participant completed two FFQs, one at the beginning (FFQ1) and one at the end of 12 months (FFQ2). During this time, twenty-four 24HRs were completed (2 each month). In addition, fasting blood and 24-hour urine samples (BioM) were also obtained each season for a total of four samples. The average of the 24HRs as well as the four serum and urine samples were used for comparison to the FFQs

 Upon entering the study, an FFQ was completed for each participant (FFQ1), and blood and 24-hour urine specimens were also collected (Figure 1). Then, for the following twelve months, two 24HRs were completed each month (total of 24 in the study period) with the biological specimens collected again each season (total of 4 specimen collections). At the end of the study (month 12), another FFQ was completed (FFQ2).

###  Food Frequency Questionnaire Design and Administration

 The PERSIAN Cohort FFQ is a 113-item, semi-quantitative FFQ, modified from two previously validated FFQs in Iran: the FFQ used in the Golestan Cohort Study (GCS) and that of the Tehran Lipid and Glucose Study (TLGS).^14,15^ We decided to use a modified version of these FFQs for two main reasons. First, given the large volume of questionnaires completed for participants upon enrolling in the cohort, both the GCS and the TLGS FFQs were too long and believed to be tiring for participants. Secondly, different forms of some foods included in the GCS and TLGS FFQs were repeated over several items, which we believed not only makes the FFQ longer to complete, but also increases overestimation of intake due to overlap between items. We therefore looked over both FFQs and modified their item lists, combining repetitive items or those we believed to be similar in nutrients, and omitting others that were either not widely used throughout Iran or those that did not contribute much energy and nutrients to the diet, reaching 113 items, that were termed the *standard* FFQ items.

 In addition to the *standard *FFQ items, we asked nutrition experts native to each of the PERSIAN Cohort locations to evaluate the FFQ and to add several *local* FFQ items, if needed. The basis for choosing the *local* food items was either foods that were used on a regular basis in that population, or were nutrient, and/or energy-dense and not already included in the FFQ. These items, which consisted of local breads, sweets and a few fruits and vegetables, were center-specific and ranged between 5‒10 items across the different cohorts.

 Our FFQ was interviewer-administered as a large proportion of the population were illiterate or had low literacy levels. All interviewers across the 18 cohort centers were trained based on the same study protocol and by the same educational team to ensure consistency across the cohorts. Interviewers asked participants about their usual intake of each food item over the previous year. The participants reported their intake as the number of times they would consume a food in one of the following four time periods: day, week, month or year. They then estimated how much of the item they consumed each time it is eaten, based on standard portion sizes pertaining to each item. Tools, such as utensils, cups, plates, food models as well as a food album picturing the FFQ portion sizes was also used by the interviewers to increase the accuracy in reporting portion sizes. These tools were also centrally purchased and distributed to the cohort centers again for consistent estimation of portion sizes. Further details about the design and administration of the PERSIAN Cohort FFQ have been previously published.^6^

###  Dietary Reference Method

 We used the 24HR as one of our reference methods to validate the PERSIAN Cohort FFQ. The 24HRs were interviewer-administered and the United States Department of Agriculture (USDA) multiple pass method was used to complete them.^16^ Based on the study protocols, the 24HRs were to be completed in person, but whenever it was not possible for the participants to attend the cohort site for this purpose, they were completed over the phone in order to limit missing 24HRs. Any individual missing more than twelve 24HRs, or all 24HRs in one season, was excluded from analysis.

 While a method with the least correlated errors to the FFQ, such as diet records, is recommended to be used as the reference method, many individuals in our study had limited reading ability or were illiterate and completing dietary records was not applicable in this population. In such instances, repeated 24HRs is the next method of choice.^5^ The 24HR is also more feasible, not requiring the high participant motivation needed to complete diet records, and is used as the reference method against FFQs by over 75% of validation studies.^17,18^ In addition, using 24HRs adjusted for within-person variation, has been shown to have similar correlations to those observed when diet records are used as a reference method.^19^

###  Nutrient Composition Analysis

 Frequency data collected by the FFQ was first converted to grams per day (g/d) as follows: the reported times per day/week/month/year was converted to times per day, then multiplied by the weight (in grams) of the portion size consumed. The *local* items added to the FFQ of each cohort center were also equated to the *standard* FFQ items based on their composition and converted to g/d. A nutrient database was then prepared using standard, non-branded foods in the USDA Food Composition Tables (USDA-FCT), containing the energy and nutrients *per one gram* of each FFQ item.^19^ This database was checked by four nutritionists to ensure that foods chosen from the USDA-FCT are the best equivalent for the FFQ items with regard to major ingredients and macronutrients. For foods not included in the USDA-FCT, such as native Iranian foods or a few of the *local* food items, the weighted average of major ingredients was used or several foods were combined to equate that food item. In the final step, to obtain the necessary nutrient data, the g/d of each item was multiplied by the energy and nutrients per one gram of the item, to acquire the energy and the amount of each nutrient consumed per day (n/day).

 The reported intake of foods recorded by the 24HRs was also converted to g/d. A few foods recorded in the 24HRs that were not included in the FFQ were combined or converted to corresponding items on the FFQ. Nutrient information from the 24HRs was obtained using the same database and procedures explained above.

###  Biomarkers

 Fasting blood was collected from 10% of participants (n = 130) four times during the study period (Figure 1) to measure serum levels of folate, cholesterol and selected fatty acids (Table 1). The average of the four measurements was calculated and used in all pertinent analyses. Serum folate (ng/L) was measured by the electrochemiluminescence immunoassay (ECLIA) and cholesterol (mg/dL) was measured by the Enzymatic Colorimetric Assay.

**Table 1:** Biomarkers Measured as Part of the PERSIAN Cohort FFQ Validation Study

	**Biomarker measured**	**Direct or grouped comparison**
Serum	Folate	Direct comparison to dietary folate
	Cholesterol	Direct comparison to dietary cholesterol (taking into consideration potential intake of cholesterol lowering medications)
	Myristate (14:0)	Grouped and compared to dietary saturated fatty acids
	Palmitate (16:0)	
	Stearate (18:0)	
	Palmitoleate (16:1n-7)	Grouped and compared to dietary monounsaturated fatty acids (MUFA)
	Elaidate (18:1n-9t)	
	Oleate (18:1n-9)	
	Linoleate (18:2n-6)	Grouped and compared to dietary Omega-6
	Gamma-linolenate (18:3n-6)	
	Dihomo-gamma-linolenate (20:3n-6)	
	Arachidonate (20:4n-6)	
	Alpha-linolenate (18:3n-3)	Grouped and compared to dietary Omega-3
	Eicosapentaenoate (EPA) (20:5n-3)	Direct comparison to dietary EPA, and grouped and compared to dietary Omega-3
	Docosahexaenoate (DHA) (22:6n-3)	Direct comparison to dietary DHA, and grouped and compared to dietary Omega-3
24-Hour urine	Urea (as an indicator of protein intake)	Calculated conversion compared to protein
	Sodium (Na)	Direct comparison to dietary sodium
	Potassium (K)	Direct comparison to dietary potassium

 As for fatty acid measurements, lipid fatty acid methyl esters were synthesized via transmethylation with methanol and acetyl chloride, neutralized, and extracted into hexane.^20^ They were stored at -20 °C under nitrogen. The methyl esters were separated on a capillary column (TR CN100, Teknokroma, Barcelona, Spain) using a Buck Scientific model 610 gas chromatograph equipped with a flame ionization detector. Peak retention times were determined using known standards, and analyzed with Peak Simple software (SRI Instruments, Torrance, CA). While 13 fatty acids were measured in serum, we did not have adequate dietary information to perform one-to-one comparisons for all of them and therefore, we grouped them into saturated fatty acids (SFA), monounsaturated fatty acids (MUFA), as well as omega-3, omega-6, docosahexaenoate (DHA) and eicosapentaenoate (EPA), comparing them with the corresponding dietary information (Table 1).

 We also collected 24-hour urine (24H-U) samples each season. Creatinine levels were measured by the Enzymatic Colorimatric Assay (Jaffe) to assess completeness of 24H-U samples.^21^ Urea, sodium and potassium measurements were obtained from the 24H-U, and again, the average of the four measurements was used in analysis. Urea was measured by photometric detection and used as an indicator of protein intake by being converted to protein using the following formula: [UUN (g/d) + 0.031 × body weight (kg)] × 0.625.^22,23^ Sodium and potassium were measured by flame photometry.

###  Statistical Analysis

 Descriptive analysis was presented as means and standard deviations (SD) for all nutrients obtained from the two FFQs and the 24HRs. Energy-adjusted (EA) nutrient intakes were estimated using energy densities (nutrient/energy). Normality assumption of all nutrients was assessed using Q-Q normal plots. Since distributions of most nutrients were far from normal, log transformation was performed to increase normality. The validity of FFQ1 and FFQ2 relative to the 24HRs was evaluated by Pearson’s correlation coefficients (PCC),presented as crude (C-PCC) and energy-adjusted (EA-PCC) correlations. To account for intra-person variability and the number of repeated measurements of the 24HR, de-attenuated energy-adjusted correlation coefficients (DEA-PCC) were obtained using the Rosner and Willett’s formula.^5^ Cross-classification analysis was conducted to determine agreement between the questionnaires, such that nutrient intakes were categorized into tertiles and the proportion of individuals classified in the same, adjacent and extreme categories was estimated.

 For serum biomarkers, to remove variation due to non-dietary factors in plasma nutrient levels, residuals from multivariable linear regression models were obtained after adjusting for age (years), sex (male, female), current smoking status (yes, no), and BMI [weight (kg)/height (m)^2^] at enrollment. Also, for nutrient intakes that were compared with biomarkers, residuals were calculated using models adjusting for the same variables. To estimate the sample correlations and validity coefficients (VC) between biomarkers, nutrient intakes estimated from FFQ2, and 24HR with true value, we used the method of triads.^24^ The correlation between the 24HR and biomarkers with the other variables was corrected for intra-person variability.^5^ We also assessed reproducibility using crude and energy-adjusted intra-class correlation coefficients (ICCs) and their 95% confidence intervals (95% CI) using random-effects analysis of variance.

## Results

 After excluding individuals who missed more than twelve 24HRs, 978 individuals remained in the study. Of these individuals, 76.5% completed more than twenty 24HRs with 63% completing all. The baseline characteristics of the study population, as well as those excluded, are shown in Table 2. There were no significant differences in the age, gender and BMI of these individuals, while those excluded were significantly more from urban areas with higher education (Table 2). The mean age of those included in the study was 46.6 ± 8.25 years and 42% were male.

**Table 2:** Baseline Characteristics of Participants in the Validation Study: Comparison of Those Included vs. Excluded Due to Missing 24HRs

		**Study Participants (N=978)**	**Participants Excluded (N=119)**	***P*** ** value**
Age (years), Mean ± SD		46.6 ± 8.25	46.2 ± 7.3	0.630
BMI (Kg/m^2^), Mean ± SD		28.3 ± 7.95	28.4 ± 4.9	0.785
Gender N (%)	Male	411 (42.0)	56 (47.1)	0.433
	Female	567 (58. 0)	63 (52.9)	
ResidencyN (%)	Urban	794 (81.2)	115 (96.6)	< 0.001
	Rural	184 (18.8)	4 (3.4)	
EducationN (%)	Illiterate or primary	419 (42.8)	43 (36.1)	0.010
	Secondary or High school	429 (43.9)	45 (37.8)	
	University Education	130 (13.3)	31 (26.1)	

###  Validity and Reproducibility of the PERSIAN Cohort FFQ against the 24HR

 To assess the validity of the PERSIAN Cohort FFQ, C-PCC, EA-PCC and DEA-PCC were obtained comparing each of the two FFQs completed (FFQ1 and FFQ2) to the 24HRs (Table 3). Total energy intake (not energy-adjusted) was moderately correlated when comparing FFQ1 to the 24HRs (DEA-PCC: 0.57), and highly correlated when FFQ2 was compared (DEA-PCC: 0.63). Macronutrient DEA-PCC are as follows in comparing FFQ1 and FFQ2 to the 24HRs, respectively: protein: 0.56 and 0.62, total lipids: 0.51 and 0.55, and total carbohydrates: 0.42 and 0.51. Caffeine, iron, fluoride, manganese, selenium, niacin, and total folate, as well as essential and non-essential amino acids showed high correlations (> 0.6) in both FFQ1 and FFQ2 comparisons, while calcium, zinc, riboflavin, and pantothenic acid had high correlations in FFQ2 vs. 24HRs only. The remaining micronutrients showed moderate (0.4‒0.6) correlations in both sets of comparisons, with the exception of sodium in FFQ1, and vitamins B6, and B12 in both FFQ1 and FFQ2, which showed poor correlations (< 0.4).

**Table 3:** Crude (C-PCC), Energy-Adjusted (EA-PCC) and Deattenuated, Energy-Adjusted (DEA-PCC) Pearson’s Correlation Coefficients Comparing FFQ1 and FFQ2 to the 24HRs to Assess Validity of the PERSIAN Cohort FFQ, and FFQ1 to FFQ2, to Assess Reproducibility of the Questionnaire

	**Validity**						**Reproducibility**	
	**FFQ1 vs. 24HRs**			**FFQ2 vs. 24HRs**			**FFQ1 vs. FFQ2**	
	**C-PCC**	**EA-PCC**	**DEA-PCC**	**C-PCC**	**EA-PCC**	**DEA-PCC**	**C-ICC (95% CI)**	**EA-ICC (95% CI)**
Energy	0.57	—	—	0.63	—	—	0.73 (0.7‒0.77)	—
Protein	0.58	0.55	0.56	0.63	0.61	0.62	0.73 (0.69‒0.76)	0.57 (0.51‒0.63)
Essential amino acids	0.6	0.6	0.62	0.68	0.66	0.68	0.76 (0.73‒0.79)	0.75 (0.72‒0.78)
Non-essential amino acids	0.62	0.63	0.64	0.69	0.69	0.7	0.76 (0.73‒0.79)	0.77 (0.74‒0.8)
Total lipid	0.56	0.50	0.51	0.60	0.54	0.55	0.7 (0.65‒0.74)	0.51 (0.43‒0.57)
Saturated fats	0.58	0.55	0.56	0.61	0.58	0.59	0.7 (0.66‒0.74)	0.54 (0.47‒0.6)
MUFA	0.5	0.45	0.46	0.6	0.55	0.56	0.69 (0.64‒0.73)	0.63 (0.58‒0.68)
PUFA	0.49	0.47	0.48	0.58	0.56	0.57	0.71 (0.67‒0.75)	0.69 (0.65‒0.73)
Total Omega-3	0.48	0.53	0.54	0.51	0.56	0.57	0.73 (0.7‒0.77)	0.76 (0.72‒0.79)
DHA	0.42	0.45	0.46	0.44	0.48	0.49	0.7 (0.66‒0.74)	0.61 (0.56‒0.66)
EPA	0.44	0.44	0.45	0.49	0.50	0.51	0.63 (0.57‒0.67)	0.63 (0.58‒0.68)
ALA	0.31	0.28	0.3	0.34	0.33	0.35	0.29 (0.19‒0.38)	0.18 (0.6‒0.29)
Total Omega-6	0.45	0.45	0.46	0.51	0.50	0.51	0.64 (0.59‒0.69)	0.63 (0.57‒0.68)
Cholesterol	0.57	0.45	0.46	0.57	0.42	0.43	0.73 (0.69‒0.77)	0.64 (0.59‒0.68)
Total Carb	0.54	0.41	0.42	0.6	0.5	0.51	0.72 (0.68‒0.76)	0.57 (0.51‒0.62)
Total sugar	0.5	0.42	0.43	0.56	0.48	0.49	0.71 (0.67‒0.75)	0.57 (0.51‒0.63)
Starch	0.56	0.56	0.57	0.53	0.52	0.53	0.70 (0.65‒0.73)	0.68 (0.64‒0.72)
Sucrose	0.55	0.52	0.53	0.62	0.57	0.58	0.71 (0.67‒0.75)	0.67 (.62‒0.71)
Glucose	0.42	0.42	0.44	0.46	0.44	0.46	0.66 (0.61‒0.70)	0.63 (0.57‒0.67)
Fructose	0.42	0.43	0.45	0.46	0.44	0.46	0.65 (0.60‒0.69)	0.62 (0.56‒0.67)
Lactose	0.47	0.47	0.49	0.53	0.53	0.55	0.74 (0.71‒0.78)	0.74 (0.71‒0.78)
Maltose	0.58	0.58	0.59	0.53	0.54	0.55	0.67 (0.63‒0.71)	0.67 (0.63‒0.72)
Galactose	0.55	0.56	0.57	0.50	0.52	0.53	0.76 (0.73‒0.79)	0.77 (0.74‒0.8)
**Micronutrients**								
Caffeine	0.73	0.71	0.73	0.73	0.7	0.72	0.79 (0.76‒0.82)	0.77 (0.74‒0.8)
Fiber	0.48	0.45	0.46	0.48	0.44	0.45	0.67 (0.62‒0.71)	0.53 (0.46‒0.58)
Calcium	0.59	0.56	0.57	0.61	0.6	0.61	0.76 (0.73‒0.79)	0.75 (0.71‒0.78)
Iron	0.61	0.62	0.63	0.62	0.62	0.63	0.76 (0.73‒0.79)	0.68 (0.64‒0.73)
Magnesium	0.55	0.45	0.46	0.59	0.51	0.52	0.7 (0.66‒0.74)	0.53 (0.47‒0.59)
Phosphorus	0.55	0.43	0.44	0.58	0.58	0.59	0.7 (0.66‒0.74)	0.63 (0.57‒0.67)
Potassium	0.53	0.49	0.51	0.57	0.57	0.59	0.67 (0.62‒0.71)	0.7 (0.66‒0.74)
Sodium	0.48	0.31	0.32	0.55	0.42	0.43	0.67 (0.62‒0.71)	0.54 (0.47‒0.6)
Zinc	0.58	0.56	0.57	0.63	0.62	0.63	0.7 (0.66‒0.74)	0.49 (0.42‒0.56)
Copper	0.49	0.48	0.49	0.55	0.54	0.55	0.67 (0.62‒0.71)	0.47 (0.39‒0.53)
Fluoride	0.75	0.72	0.74	0.73	0.68	0.7	0.79 (0.76‒0.82)	0.76 (0.73‒0.79)
Manganese	0.59	0.6	0.61	0.63	0.62	0.63	0.75 (0.71‒0.78)	0.74 (0.70‒0.77)
Selenium	0.68	0.69	0.7	0.68	0.68	0.69	0.8 (0.77‒0.83)	0.78 (0.75‒0.81)
Vitamin A (IU)	0.54	0.5	0.52	0.50	0.51	0.54	0.71 (0.67‒0.75)	0.68 (0.64‒0.72)
Beta carotene	0.56	0.53	0.55	0.51	0.51	0.53	0.71 (0.67‒0.75)	0.68 (0.63‒0.72)
Vitamin E	0.54	0.51	0.53	0.54	0.52	0.54	0.65 (0.6‒0.69)	0.45 (0.35‒0.52)
Vitamin D (mcg)	0.48	0.48	0.49	0.51	0.50	0.51	0.7 (0.66‒0.75)	0.7 (0.65‒0.74)
Cryptoxanthin	0.45	0.47	0.49	0.43	0.44	0.46	0.72 (0.68‒0.75)	0.72 (0.68‒0.75)
Lycopene	0.45	0.37	0.4	0.42	0.39	0.42	0.57 (0.51‒0.63)	0.52 (0.45‒0.58)
Lutein	0.51	0.48	0.5	0.49	0.5	0.52	0.64 (0.59‒0.69)	0.61 (0.55‒0.66)
Vitamin C	0.44	0.41	0.43	0.44	0.41	0.43	0.68 (0.63‒0.72)	0.65 (0.61‒0.7)
Thiamin	0.55	0.55	0.56	0.58	0.58	0.59	0.75 (0.71‒0.78)	0.65 (0.61‒0.7)
Riboflavin	0.57	0.56	0.57	0.60	0.6	0.61	0.73 (0.68‒0.76)	0.54 (0.48‒0.6)
Niacin	0.62	0.63	0.64	0.64	0.64	0.65	0.77 (0.74‒0.8)	0.71 (0.67‒0.74)
Pantothenic acid	0.55	0.54	0.55	0.61	0.6	0.61	0.76 (0.72‒0.79)	0.75 (0.72‒0.78)
Vitamin B6	0.34	0.3	0.32	0.38	0.36	0.38	0.34 (0.27‒0.44)	0.27 (0.16‒0.36)
Total Folate	0.63	0.66	0.67	0.65	0.67	0.68	0.78 (0.75‒0.81)	0.79 (0.76‒0.81)
Vitamin B12	0.28	0.24	0.25	0.4	0.37	0.39	0.53 (0.46‒0.59)	0.49 (0.41‒0.55)
Choline	0.58	0.44	0.45	0.61	0.52	0.53	0.69 (0.64‒0.73)	0.5 (0.43‒0.56)
Vitamin K	0.52	0.49	0.51	0.52	0.52	0.54	0.68 (0.64‒0.72)	0.63 (0.58‒0.68)

 In comparing FFQ1 and FFQ2 to assess the reproducibility of the questionnaire, C-ICC showed high correlations for energy intake (0.73). Crude correlations for protein, total lipid and total carbohydrates were also high (0.73, 0.73, and 0.7, respectively), but when they were adjusted for energy, they became moderately yet still acceptably correlated (0.57, 0.51, and 0.57, respectively). Of the 30 micronutrients assessed, 19 showed high EA-ICC (> 0.6), with only one showing poor correlations (vitamin B6).

Table 4 shows the cross classification of FFQ1 and FFQ2 vs. the 24HRs (validity), as well as FFQ1 vs. FFQ2 (reproducibility). On average, 50.9% of individuals were classified in the same category for the various nutrients when FFQ1 was compared to the 24HRs, and 53.1% when FFQ2 was compared. Only 9.1% and 7.8% of individuals were misclassified in FFQ1 and FFQ2 vs. 24HRs, respectively. When FFQ1 was compared to FFQ2, 54.1%, 38.3% and 7.6% were classified in the same, adjacent, and extreme categories, respectively.

**Table 4:** Percent Agreement for Tertiles Between FFQ1 and FFQ2 vs. the 24HRs (Validity), as well as FFQ1 vs. FFQ2 (Reproducibility)

**Nutrients**	**Validity**						**Reproducibility**		
	**FFQ1 vs. 24HR**			**FFQ2 vs. 24HR**			**FFQ1 vs. FFQ2**		
	**Same**	**Adjacent**	**Extreme**	**Same**	**Adjacent**	**Extreme**	**Same**	**Adjacent**	**Extreme**
Energy	56.1	38	5.9	55.3	39.2	5.5	58.7	36.8	4.5
Protein	54.6	38.2	7.2	52.3	40.5	7.1	55.4	37.2	7.4
Essential amino acids	53.8	39.1	7.1	59.2	37.1	3.7	56.5	36.6	6.9
Non-essential amino acids	56.9	37.2	5.8	61.3	35.1	3.6	59.7	34.6	5.7
Total lipid	49.8	41.3	8.9	52.1	40.8	7.1	55.3	37.2	7.6
Saturated fats	53.7	39.4	6.9	55	40.4	4.6	57.5	36	6.5
MUFA	47.9	41.5	10.6	53.3	38.7	8	51.9	38.2	9.9
PUFA	47	42.1	10.9	52.8	39.2	8	51.1	41	7.9
Total Omega-3	52	41.1	6.9	55.3	36.7	8	57.7	37.4	5
DHA	50.6	39.1	10.3	51.4	38.1	10.6	55.6	38	6.4
EPA	48.1	40.7	11.1	49.3	42	8.7	49.1	41.7	9.2
ALA	40.6	45.3	14.1	45.7	44.2	10.1	45.5	39.9	14.6
Total Omega-6	49.3	39.6	11.1	51.1	40.9	8	48.8	42	9.2
Cholesterol	47.5	41.8	10.7	47.9	41.6	10.5	51.4	39.2	9.3
Total Carb	46.7	42.3	11	51.5	38.9	9.6	51.4	39.4	9.2
Total sugars	45.3	42.8	11.9	48	40.9	11.1	51.1	41.7	7.2
Starch	52.4	37.5	10.1	50	40.4	9.6	52.1	38.8	9.1
Sucrose	53.4	38.2	8.4	56.5	36.9	6.5	57.1	36.6	6.3
Glucose	45.9	42.1	12	50.9	39.8	9.4	53	39	8
Fructose	46	43.1	10.9	51.8	39	9.1	52	38.8	9.2
Lactose	51.3	37.9	10.8	51.2	39.6	9.2	56.9	37.6	5.4
Maltose	52.6	37.1	10.3	48	42.7	9.2	52.4	37.8	9.8
Galactose	50.5	41.8	7.8	50.4	40.9	8.8	57.8	36.9	5.3
**Micronutrients**									
Caffeine	57.7	36.5	5.8	59.9	36.2	4	56.2	37	6.9
Fiber	48.9	40.5	10.6	49.3	39.7	11.1	53	40.3	6.7
Calcium	51.7	40.8	7.4	58.7	35	6.3	55.3	36.8	7.9
Iron	60.3	34	5.6	60.5	35.1	4.4	59.9	35.4	4.7
Magnesium	49.4	40	10.6	51.1	39.8	9.1	51.2	42	6.7
Phosphorus	46.7	42.3	11	54.3	38.5	7.1	50.9	39.9	9.2
Potassium	50.4	41.8	7.8	54.6	38.5	6.9	53.2	39.3	7.4
Sodium	45.8	41	13.1	49	40.5	10.5	46.6	41.7	11.7
Zinc	53	39.9	7.1	55.5	38.4	6	56	38.5	5.5
Copper	49.1	42	8.9	53.4	37.7	8.9	56.3	38	5.7
Fluoride	55.4	38.1	6.6	57.9	37.3	4.8	55	36.8	8.3
Manganese	56.3	36.3	7.4	58.7	35	6.3	57.6	35.7	6.7
Selenium	62.6	33.9	3.5	62.1	34.2	3.7	62.9	32.3	4.7
Vitamin A (IU)	50.8	42.2	7	53.2	39	7.8	54.3	38.9	6.9
Beta carotene	52.4	40	7.6	51.6	41.1	7.3	55.1	38.4	6.5
Vitamin E	51.5	40.9	7.5	51.8	40	8.1	53.4	39.6	7.1
Vitamin D (mcg)	49.8	40.7	9.4	51	40.1	8.9	56.6	35.5	7.9
Cryptoxanthin	45.4	44.3	10.3	48.2	42.1	9.7	57.1	36.2	6.7
Lycopene	44.4	42.3	13.3	49.6	41.6	8.7	47.6	41.3	11.1
Lutein	47.6	43.6	8.8	50.9	41.7	7.4	54.3	39.1	6.6
Vitamin C	46.2	41.8	12	47.3	41.6	11.1	53.3	37.9	8.7
Thiamin	57.5	36.3	6.3	57.6	36	6.4	57	37.2	5.8
Riboflavin	57.2	36.1	6.8	56.3	37.2	6.5	58.6	36.2	5.2
Niacin	60.4	34	5.5	60.5	33.9	5.7	58.1	36.8	5.1
Pantothenic acid	52.6	38.4	9	54.9	38.4	6.7	54.3	39.5	6.3
Vitamin B6	41.4	44.6	14	45.1	45	10	45.3	40.6	14
Total Folate	60	36.1	3.9	61.8	34.6	3.6	59.6	36.2	4.1
Vitamin B12	42.1	43.2	14.7	44.7	44.1	11.2	46.9	40.2	12.9
Choline	49.4	40.1	10.5	53	38.8	8.3	49.8	40.1	10
Vitamin K	49.5	42.6	7.8	52.8	39.7	7.5	52	40.9	7.1

###  Method of Triads Comparing the FFQ, 24HRs and Biomarkers

**Figure 2:**
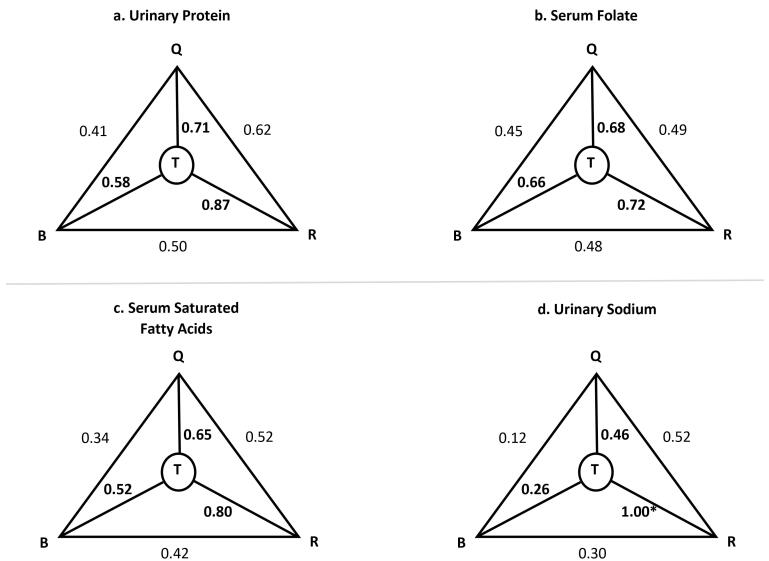
Triad Diagrams of Selected Biomarkers. Q, FFQ; B, Biomarkers; R, 24HRs; T, Truth—the validity coefficients. * Validity coefficients higher than 1 were set to 1

 We invited 140 individuals to take part in the biological sample part of our study, of whom 130 (93%) agreed. Serum and 24H-U samples were collected from each individual each season. All participants completed serum sample collections and were included in the serum biomarker analyses performed. As for the 24H-U samples, however, 46 individuals were excluded from the protein measurement analyses for two reasons: either they had an incomplete sample, or the laboratory technician did not record the total urine volume, making some urinary measurements unusable. Validity coefficients obtained by the method of triads for all biomarkers are shown in Table 5 and the triad diagram for selected biomarkers are shown in Figure 2. The lower limit, defined as the correlation between the FFQ and the biomarkers (BQ), and the upper limit, defined as the validity coefficient of the FFQ (VC_Q_) were 0.45‒0.68 for folate, 0.34‒0.65 for SFA, 0.41‒0.71 for protein, and 0.12‒0.46 for sodium. All VC_Q _were above 0.4, the accepted correlation value in the triad method. Interestingly, correlations for sodium, that were poor when the FFQ and the 24HRs were compared, were acceptable (0.46) when the method of triad was used.

**Table 5:** Validity Coefficients Obtained by the Method of Triads

		**Correlations**			**Validity Coefficients**		
		**BR**	**BQ**	**QR**	**VC**_B_	**VC**_R_	**VC**_Q_
Serum^a^	Folate	0.48	0.45	0.49	0.66	0.72	0.68
	Cholesterol	0.51	0.43	0.39	0.75	0.68	0.57
	SFA	0.42	0.34	0.52	0.52	0.80	0.65
	MUFA	0.4	0.29	0.5	0.48	0.83	0.60
	Omega-3	0.34	0.33	0.41	0.52	0.65	0.63
	Omega-6	0.32	0.29	0.53	0.42	0.76	0.69
	DHA	0.51	0.38	0.40	0.70	0.73	0.54
	EPA	0.26	0.27	0.44	0.40	0.65	0.67
24-Hour Urine	Protein	0.5	0.41	0.62	0.58	0.87	0.71
	Sodium (Na)	0.3	0.12	0.52	0.26	1.00*	0.46
	Potassium (K)	0.32	0.14	0.55	0.29	1.00*	0.49
	Na:K	0.33	0.22	0.52	0.37	0.88	0.59

BR, Correlation between the biomarkers and 24HRs; BQ, Correlations between the biomarkers and FFQ; QR, Correlations between the FFQ and 24HRs. VCB, Validity coefficient of the biomarkers, VCR validity coefficient of the mean of the 24HRs, VCQ validity coefficient of FFQ2 ^a^ Serum measurements have been adjusted for age, gender, BMI, smoking status at the time of sample collection (yes/no). * Validity coefficients higher than 1 were set to 1. Lower limit is QR while the upper limit is VCQ.

## Discussion

 FFQs are the most commonly used tool to assess dietary intake in longitudinal epidemiological studies. Completing FFQs is inexpensive and imposes a small burden on respondents, compared to other dietary methods^5,25,26^ The FFQ however, relies on memory and intake of foods is estimated based on usual intake; therefore, the data it gathers should not be used as absolute intake measures, but instead, it should be used to rank individuals based on their reported intakes.^5^ In order to ensure that an FFQ acceptably measures what it is intended to measure, it needs to be validated first. In this study, we evaluated the validity and reproducibility of the PERSIAN Cohort FFQ in assessing nutrient intakes of respondents, against multiple 24HRs and selected serum and urinary biomarkers and found it to be acceptable to rank individuals based on their nutrient intake.

###  Validity and Reproducibility Assessment

 We used PCC to assess validity between the two questionnaires. Energy intake showed moderate (0.57) and high (0.63) correlations when FFQs 1 and 2 were compared to the 24HRs, respectively. Given that correlation coefficients can be attenuated by random measurement errors (due to high day-to-day variation in food intake recorded by the dietary recalls), experts have suggested correlations to be corrected by removing the effect of the random measurement errors, in order to estimate the true correlation between two variables. We, therefore, also calculated the DEA-PCC for all macro- and micronutrients and consider this estimate to be the best indicator of our FFQ’s validity for nutrient intake.

 Among the macronutrients, protein had the highest DEA-PCC followed by total lipids and total carbohydrates, in both comparisons, all showing acceptable correlations (protein: 0.56‒0.62, total lipids: 0.51‒0.55, total carbohydrates: 0.42‒0.51). While adjusting for energy and de-attenuating the correlations slightly affected protein and fat intake, carbohydrates were much more greatly affected, and crude measures changed from 0.54 to 0.42 (FFQ1 vs. 24HRs) and from 0.6 to 0.51 (FFQ2 vs. 24HRs). Given that grains, mostly eaten as breads and rice, are the main staple food in Iran, it was expected that carbohydrates would be more considerably affected by these energy-adjustments.

 We also observed acceptable correlations upon further breakdown of macronutrients, with essential and nonessential amino acids showing high correlations (0.62‒0.7) and SFA, MUFA, PUFA and other essential lipids showing moderate DEA-PCC (0.46‒0.59). Only alpha-linoleic acid (ALA) had a poor correlation (0.3‒0.35). Correlations for total sugar, as well as the various types of carbohydrates, ranged from 0.43 to 0.59, which are also considered acceptable.

 Comparing to the results obtained by the two previously validated FFQs in Iran, in the GCS study, correlations for energy ranged from 0.56‒0.62 for the four FFQs completed, while protein, fat and carbohydrates ranged from 0.49‒0.73, 0.44‒0.58 and 0.51‒0.66, respectively.^14^ While our FFQ showed better correlations for protein and fat, the GCS FFQ estimated carbohydrate intake better. In the TLGS study, the adjusted PCC obtained were as follows: energy, 0.46 in women and 0.55 for men; protein, 0.5 in women and 0.65 in men; fats, 0.38 in women and 0.59 in men; and carbohydrates, 0.47 in women and 0.39 in men.^15^

 About 30 micronutrients were also compared between the FFQs and the 24HRs. When FFQ1 was compared to the average of twenty-four 24HRs, DEA-PCC ranged from 0.25‒0.74, with the lowest correlation belonging to vitamin B12 and the highest to fluoride. DEA-PCC ranged from 0.38‒0.72 in FFQ2 vs. 24HRs, where the lowest DEA-PCC was observed for vitamin B6 and the highest for caffeine. Vitamin B6 and caffeine were the second lowest and second highest correlations in FFQ1 vs. 24HRs, respectively. Important micronutrients, such as iron (0.63, 0.63), calcium (0.57, 0.61), potassium (0.51, 0.59), zinc (0.57, 0.63), folate (0.67, 0.68) and vitamins A (0.52, 0.54), E (0.53, 0.54), and C (0.43, 0.43) all had moderate to high correlations in both FFQ1 and FFQ2 comparisons to 24HRs, respectively. Of the B vitamins, only vitamins B6 (0.32, 0.38) and B12 (0.25, 0.39) showed poor correlations.

 Previous studies showed adjusted correlations ranging from 0.11 for beta-carotene to 0.71 for phosphorus and 0.21 for zinc to 0.71 for energy (Iran), 0.14 for selenium to 0.77 for alcohol (New Zealand), 0.02 for iodine to 0.55 for manganese (Poland), 0.06 for iron to 0.62 for fiber (United Arab Emirates), -0.07 for vitamin D to 0.89 for protein (Peru), -0.03 for riboflavin to 0.41 for % energy from carbohydrates (Tanzania), 0.09 for energy to 0.85 for animal protein (USA and Canada), -0.17 for fluorine to 0.64 for sodium (Qatar), -0.002 for vitamin A to 0.34 for carbohydrates and 0.78 for sodium to 0.99 for carbohydrates (Lebanon), 0.24 for fiber to 0.93 for total MUFA (Morocco), 0.24 for vitamin C to 0.46 for carbohydrates (Malaysia), 0.1 for total fat to 0.8 for vitamin A (Ethiopia), 0.14 for protein to 0.44 for fat (Chile), 0.21 for energy to 0.84 for caffeine (USA), 0.54 for fiber to 0.86 for alcohol (Germany), and 0.36 for sodium to 0.77 for ethanol (Mexico).^15,25-41^ While the results for the lowest and highest correlations vary among different studies, our results are similar to most previously validated FFQs.

 Over 50% of our participants were classified in the same tertiles of nutrient intake and less than 10% were on average misclassified into the extreme category. These figures are similar to those of previous studies and are generally considered as good agreement between the two questionnaires.^26,42^

 The method of triads was used to compare biomarker levels to the FFQ. Generally, a validity coefficient above 0.4 is considered acceptable^24^ and all VC_Q_ in our study were above this level. While many studies also measure serum levels of calcium, sodium, beta-carotene and retinol, we did not measure these biomarkers for several reasons. The serum levels of these biomarkers, especially beta-carotene and retinol are highly sensitive to light and become undetectable if the samples undergo freeze-defreeze or if the appropriate storage methods are not employed. Given that we collected sample from seven locations but decided to perform the analyses in one location in Tehran, we required biomarkers that were more stable, in case the samples became exposed to undesirable conditions. Also, these biomarkers are subject of strong homeostatic regulations and some are affected by many other factors in the body, such as availability of binding proteins and therefore, their concentrations may not best represent that of intake.^5^ Fatty acid levels, on the other hand, besides being highly stable in serum, have been shown to be related to all-cause mortality and cardiovascular death in previous studies, which is an important indicator in cohorts focusing on NCDs.^43^ Folate intake is also strongly predictive of folate levels and is suggested as a biomarker for validation studies.^5^

 To assess reproducibility, intraclass correlation coefficients were obtained between FFQ1 and FFQ2. Moderate (0.4‒0.6) to highly acceptable ( > 0.6) reproducibility was observed for energy, macro- and micronutrients. These findings are comparable to those of other studies.^14,15,25,26,29,35,41^

## Strengths and Limitations

 One strength of our study is that it is multi-center, including an adequate sample size from different regions of Iran with varying eating habits. While our intention was not to validate the FFQ for each center separately, we included the various centers to ensure that they are represented in the validation study so that we can presume the FFQ to be adequate for use in different Iranian populations. This makes our FFQ different from those previously validated in Iran.

 The 24HR has correlated errors with the FFQ, which can be regarded as a limitation. Experts suggest choosing a reference method in validation studies, whose measurement errors are independent and uncorrelated from those of the FFQ, and the diet record is the primary method of choice among the various dietary questionnaires. However, the diet record requires high cooperation from respondents as well as adequate literacy levels. Given that a considerable proportion of our population were illiterate or had low literacy levels, the interviewer-administered 24HRs were the most adequate choice for reference method.^5^ This method is the most commonly used method across other validations studies, as well, due to its feasibility.^18^ We did complete twenty-four 24HRs for each person, on the other hand, which is a strength of our study, well-capturing day-to-day and seasonal variations in food intake. Also, our recorded intakes were adjusted for intra-person variations, making them comparable to data collected by diet records.^19^

 In addition, to overcome the limitation of correlated errors that the 24HRs have, we measured several biomarkers which have the fundamental advantage of being uncorrelated with the FFQ.^5^ We tried to measure biomarkers that can be regarded as representative of intake, such as folate, fatty acids, and urinary sodium, potassium and nitrogen.

 The fact that about 35% of individuals were excluded from the 24H-U sample protein analysis is, however, a weakness of our study. Most of these exclusions occurred because lab technicians at two centers had not recorded the overall urine volume and measurement of creatinine and urea was not possible without this value. The number of samples we ended up with (n = 84) is still about 10% of our total population, which is in-line with the sample size suggested by experts.^5^ And again, this weakness only pertains to the protein measurements and other urinary measurements are acceptable, as they were measured as quantities in liters without the need to incorporate the total urine volume in any formula or measurement.

 We used the USDA-FCT to assess the nutrient intake of the PERSIAN Cohort participants because the food composition table specific to Iranian foods is very limited in the number of foods and nutrients assessed. Therefore, it is very important not to use our estimates of nutrient intake as actual amounts consumed. This is especially important for nutrients that are dependent on soil in various regions such as selenium, zinc or iodine.^44-46^

## Conclusion

 We evaluated the validity and reproducibility of the PERSIAN Cohort FFQ in assessing nutrient intakes, using 24HRs as well as serum and urinary biomarkers, finding it acceptable and comparable to previously validated FFQ. Data gathered from this FFQ should be used to rank individuals based on their intake, instead of measuring absolute intake. Ranked data can be acceptably used in future studies of diet-disease associations.
